# Stochastic variation in foraging traits within inbred lines of *Drosophila*

**DOI:** 10.1371/journal.pone.0289864

**Published:** 2025-01-16

**Authors:** Kaiya Hamamichi, Yuma Takahashi

**Affiliations:** 1 Graduate School of Science and Engineering, Chiba University, Chiba, Japan; 2 Graduate School of Science, Chiba University, Chiba, Japan; National Cheng Kung University, TAIWAN

## Abstract

Investigating the causes and consequences of niche partitioning in populations is a major goal in ecology and evolutionary biology. Previous studies have investigated genetic and environmentally induced variation in resource utility and their ecological implications. However, few studies have explored variability (non-genetic, stochastic variation) as a factor contributing to variation in resource utility. In this study, we studied the variability in foraging traits of *Drosophila lutescens*, a species of wild fruit fly. Using 70 iso-female lines from a single population, we observed two foraging traits, i.e., locomotive speed and resource preferences, in an “8”-shaped experimental arena containing different types of fruit juices. The mean locomotive speed and relative preference for orange juice over grape juice varied significantly among iso-female lines. Additionally, the degree of intraline variation (variability) was detected a fold-change of larger than 2-fold between the smallest line and the largest line. While the mean locomotive speed itself did not correlate with mean resource preferences, the variability of locomotive speed significantly correlated with that of resource preferences. These results suggest that the degree of variability within inbred lines for both locomotive activity and resource preference is potentially partly genetic and that a shared genetic basis may govern variability in these traits. The variability of a particular trait is considered to interact cooperatively with the variability of several other traits in creating phenotypic intraspecific variation within a population.

## Introduction

Niche and resource partitioning play a crucial role in ecology, enhancing community, and ecosystem functioning, stability, and productivity [1–4]. Niche partitioning effectively reduces potential competition among individuals [5], and niche width reflects resource abundance along the resource axis, the pattern of intraspecific competition, and elements of natural selection [6]. In recent decades, the ecological function of intraspecific resource partitioning has garnered increasing attention, with studies demonstrating the influence of niche partitioning, even within a single species, on population dynamics [7–10]. Therefore, understanding resource and niche partitioning within a population is essential for comprehending population dynamics [11–14].

Phenotypic variation between individuals can arise from three causative factors: genetic variation, environmental variation (phenotypic plasticity), and stochastic variation (referred to here as “variability”) [15]. The study of genetic and environmental variation in the context of intraspecific resource partitioning has been well-documented [16–20]. For example, empirical laboratory experiments have provided evidence of a causal link between genetic differences and variation in resource preferences [21]. Additionally, it has been established that the dietary preferences of the tobacco hornworm *Manduca sexta* are subject to plasticity and determined based on the previously consumed plant [22, 23]. On the other hand, developmental noise, i.e., random fluctuations during development, is one of the factors producing variability. Variability can contribute to variation among individuals sharing the same genetic and environmental background [24]. However, few studies have explored variability in foraging behavior and resource use within populations. To comprehensively understand the mechanisms underlying intraspecific resource partitioning, both genetic variation and variability contributions must be thoroughly investigated.

In *Drosophila*, the presence of stochastic behavioral variation among individuals and its underlying neural mechanisms have been reported [25]. Here, we aim to demonstrate the interline variation, focusing on the mean and variation (which are likely due to genetic differences and variability, respectively) in foraging traits in the wild fruit fly *Drosophila lutescens*. We established iso-female lines using females from a single natural population and evaluated the mean and variation in two essential foraging traits, namely locomotive activity and resource preferences, which shape resource partitioning. Subsequently, we estimated the heritability of each trait, followed by an examination of the genetic correlation between the variability for each trait.

## Materials and methods

### Sampling and study species

*Drosophila lutescens*, a member of the *melanogaster* group within the subgenus *Sophophora*, is considered a generalist species [26]. To explore genetic variation and variability within the same genotype, we used multiple independent isofemale lines derived from the same population in the present study. Iso-female line is a laboratory line which is derived from a single, wild-caught female. Typically, the offspring are allowed to reproduce over several generations to reduce genetic heterogeneity. To establish the iso-female lines of *D*. *lutescens*, the females were collected from the campus of Chiba University (35°37′34″ N, 140°6′9″ E) from January 14 to April 2 (spring) and October 5 to November 9 (autumn), 2020 (S1 Table). The iso-female lines used in our study were expected to exhibit season-specific genetic and phenotypic changes because many studies on *Drosophila* have suggested that significant genetic and phenotypic changes occur across seasons due to changes in allele frequency in a small number of adaptive genes [27–29]. Note that our previous genome-wide population genetic analysis indicated that the seasonal populations (spring and autumn) are genetically indistinguishable [30]. Each collected female was cultured in a plastic vial (30 mm in diameter and 100 mm in height) with a nutritive medium following the protocol of Fitzpatrick et al. [31] to establish iso-female lines. The composition of the medium was as follows: 500 ml of H_2_O, 50 g of sucrose, 50 g of active yeast, 6.5 g of agar, 5.36 g of KNaC_4_H_4_O_6_·4H_2_O, 0.5 g of KH_2_PO_4_, 0.25 g of NaCl, 0.25 g of MgCl_2_, 0.25 g of CaCl_2_, and 0.35 g of Fe_2_(SO_4_)·6.9H_2_O. Throughout the study, we used the same medium and vials for maintaining the lines. All lines were raised in a 12L12D light-dark cycle and maintained on homogeneous medium. Each line was inbred in en masse for at least 20 generations. To ensure age homogeneity, individuals aged 1–7 days old after emergence were used in all experiments. These experiments were conducted in 2021–2022, using 70 established iso-female lines.

### Quantification of variation in foraging traits and heritability

**Fig 1:**
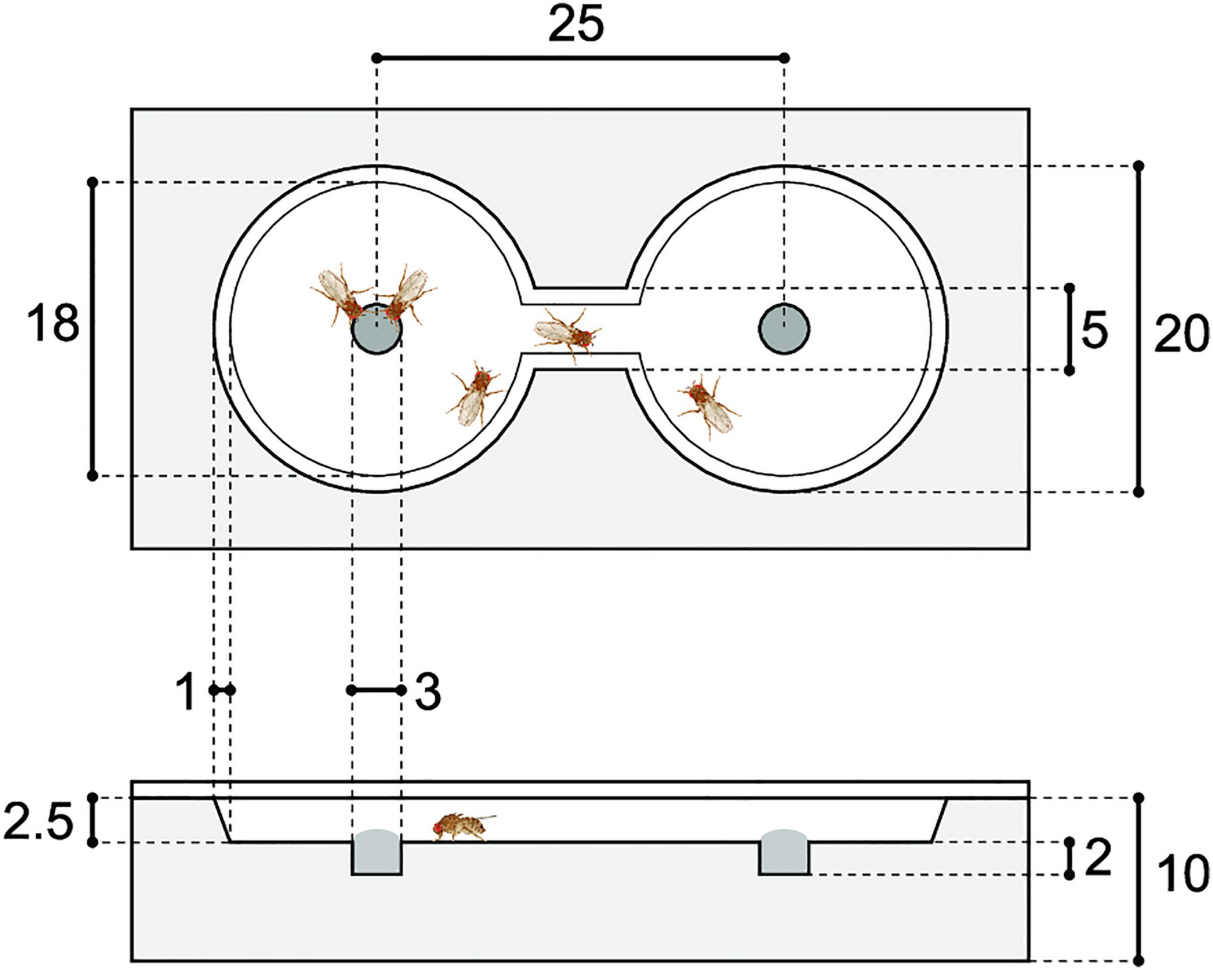
Anterior (upper) and transverse (lower) sections of the experimental arena. Black circles represent resources, whereas white regions indicate the area where individuals can walk. Unit of length: mm.

To quantify foraging traits, an “8”-shaped experimental arena with two confined chambers and a connecting passageway was used (Fig 1). To investigate feeding preferences, we used commercially available 100% orange juice and grape juice, both of which are readily available. In *Drosophila melanogaster*, it is known that they exhibit different preferences for orange and grape, suggesting that fruit flies can distinguish between the odors of these two fruits [32, 33]. Either orange juice or grape juice was placed in the central indentation of each of the two chambers. The position of the two types of juice was randomized for each observation. Five females per iso-female line were introduced into the arena after being anesthetized using CO_2_. The flies were placed at the center of the passageway to minimize any initial position bias. Subsequently, the arenas were covered with slide glass, placed in an incubator (25°C), and recorded via a camera for 66 min. To mitigate the effects of anesthesia, we extracted coordinate data through automated tracking (FlyTracker in MATLAB) from the last 44 min of the video. Twenty-five to 40 replicates (individuals) were used per line.

Resource preferences were estimated using the preference index (*PI*), calculated based on the time spent in the orange and the grape feeding chamber. *PI* was calculated as follows: *PI* = log_10_(*O*/*G*), where *O* and *G* represent the time spent in the orange and grape feeding chambers, respectively. A positive *PI* value indicated a preference for orange, whereas a negative value indicated a preference for grape. A *PI* value of 0 indicated no preference between the two.

To assess foraging activity, locomotive activity was quantified by calculating the average locomotive speed while searching. Data were excluded if the locomotive speed was <0.01 mm/s, which indicated that the fly had reached the food (3.5 mm from the center of the food). The locomotive activity was measured as the distance traveled every ~0.67 s.

Phenotypic variation within a line maintained under constant conditions was assumed to represent the degree of variability, and was measured as the standard deviation (SD) for the two traits. The coefficient of variation (%CV) for locomotive activity was also calculated to demonstrate robustness across different indices of variation. Note that %CV for *PI* was not used because the phenotypic values of *PI* crossed zero, causing lines with values near zero to be considered outliers. The %CV for locomotive activity was calculated as the ratio of the SD to mean locomotive speed as follows: SD/Mean × 100. To examine the correlation between resource preferences and locomotive activity, we conducted a Spearman’s correlation test. The covariance between the two variables was also calculated.

The heritability of each trait was evaluated using variance component analyses (VCA), with each trait of each individual assigned as an objective variable, and the line and age assigned as random effects.

### Statistical analyses

All statistical analyses were conducted using R software (version 4.2.2). The R package *R*.*matlab* was used to read the coordinate data obtained from video tracking. To examine the mean of each trait among lines, we used linear mixed effects models with restricted maximum likelihood (REML) and a likelihood ratio test. The models were as follows: *Y* ~ *L*_random_ + *A*_random_, where *Y* is each trait of each individual, *L*_random_ is the effect of the line treated as random, and *A*_random_ is the effect of age as random. Note that we chose not to include the arena as a random effect for two reasons. First, each arena contained individuals of only a single age class and line, resulting in a high correlation between the arena factor and both age and line. Multicollinearity has been shown to negatively impact the precision of estimates. As our primary focus is on the effect of line, we opted not to include the arena factor. The second reason is the large number of arenas relative to the small number of replicates within each arena. Because the number of replicates per cluster of random effects was only 5 in this study, statistical power is suggested to decrease with a reduction in the number of replicates per cluster of random effects [34]. We estimated line means and their standard errors using these models. Moreover, the difference in the mean of each trait among lines was also examined by the Kruskal-Wallis test, where each trait was assigned as an objective variable, and the line was assigned as an explanatory variable. We compared the mean of each trait between sampling seasons by using *t*-tests. We also tested the deviation of the preferences in each line from zero (no preference) using binomial tests. The deviation of mean preference of 70 lines was tested by one-sample *t*-tests. Since the variance for line means might be sensitive to outlier values, we adjusted the variance estimations using jackknife estimation with the equation as follows:

$${\hat{\theta }}_{jack}=n\hat{\theta }-\left(n-1\right){\hat{\theta }}_{\left(.\right)}$$

$${\hat{se}}_{jack}=\sqrt{\frac{n-1}{n}\sum_{i=1}^{n}{\left({\hat{\theta }}_{\left(i\right)}-{\hat{\theta }}_{\left(.\right)}\right)}^{2}}$$

where ${\hat{\theta }}_{jack}$ is the SD of each trait adjusted using jackknife estimation, *n* is the number of replicates for each line, $\hat{\theta }$ is the SD using all individuals in each line, ${\hat{\theta }}_{\left(.\right)}$ is the mean SD estimated by repeatedly calculating it *n* times when a certain individual was eliminated, ${\hat{se}}_{jack}$ is the standard error of each trait estimated by using jackknife estimation, and ${\hat{\theta }}_{\left(i\right)}$ is the SD estimated by eliminating a certain individual. In the analysis, except when calculating %CV, locomotive speed was log-transformed to approximate a Gaussian distribution. Spearman’s correlation test was used to examine correlations between locomotive speed and *PI*, and between the SD or %CV of locomotive speed and SD of *PI*.

### Ethics statement

There are no ethical quandaries associated with the non-invasive study of an invertebrate species. This species is not an endangered or protected species. All individuals were collected within our affiliated research facility and did not necessitate permission.

## Results

**Fig 2:**
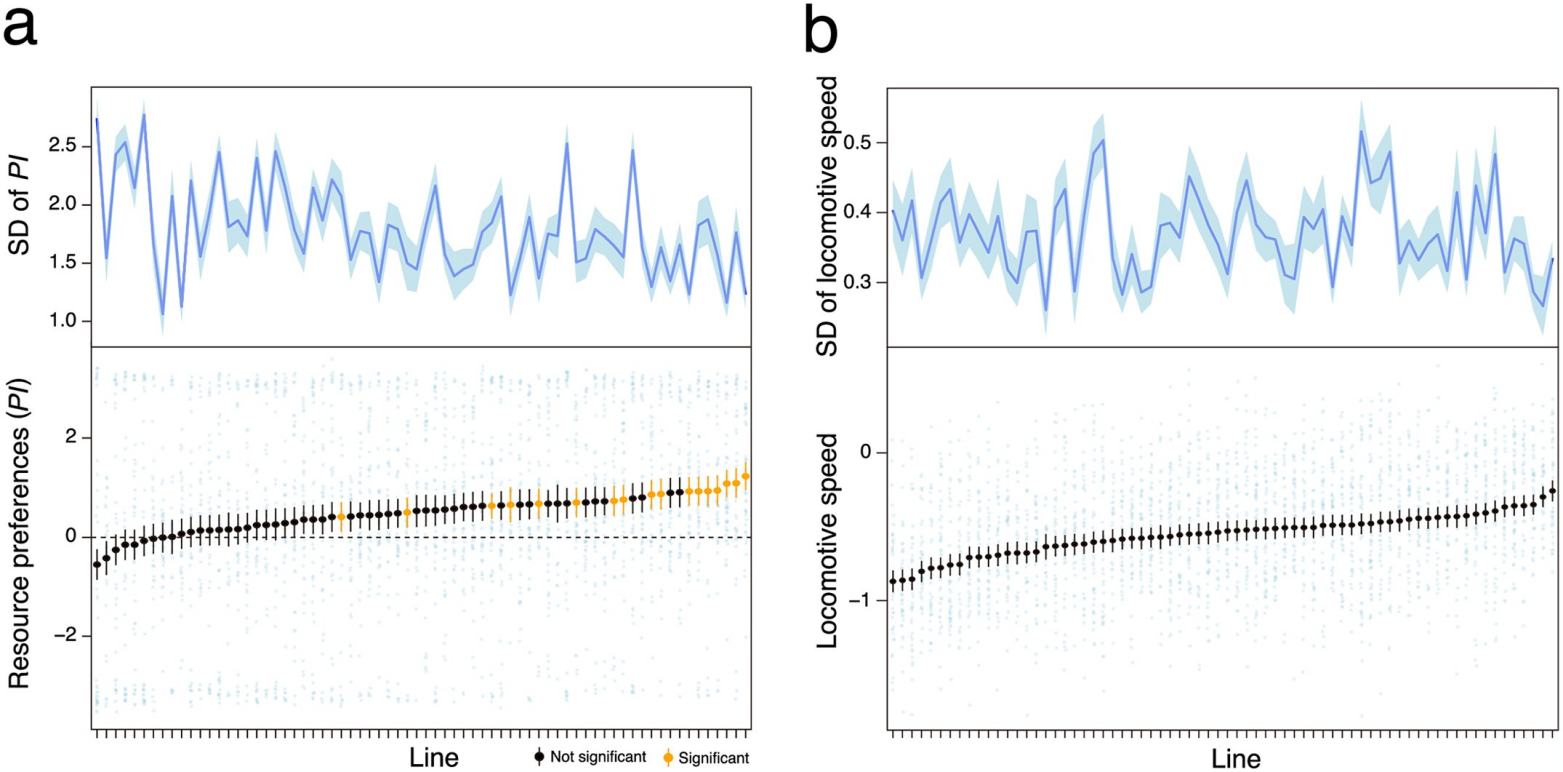
Mean (lower) and intraline variation (upper) for *PI* (a) and locomotive speed (b). Lines are arranged in ascending order based on their respective means, and error bars and ribbons (estimated by jackknife estimation) represent standard errors. The error bars and ribbons are calculated using 25–40 points. The color of the resource preference mean points indicates the results of the binomial test for binarized values (S1 Table). *P*-values were corrected using the FDR. Kruskal-Wallis test: *df* = 69, χ^2^ = 106.1, *P* = 0.0027 (a); *df* = 69, χ^2^ = 396.5, *P* < 0.001 (b).

**Fig 3:**
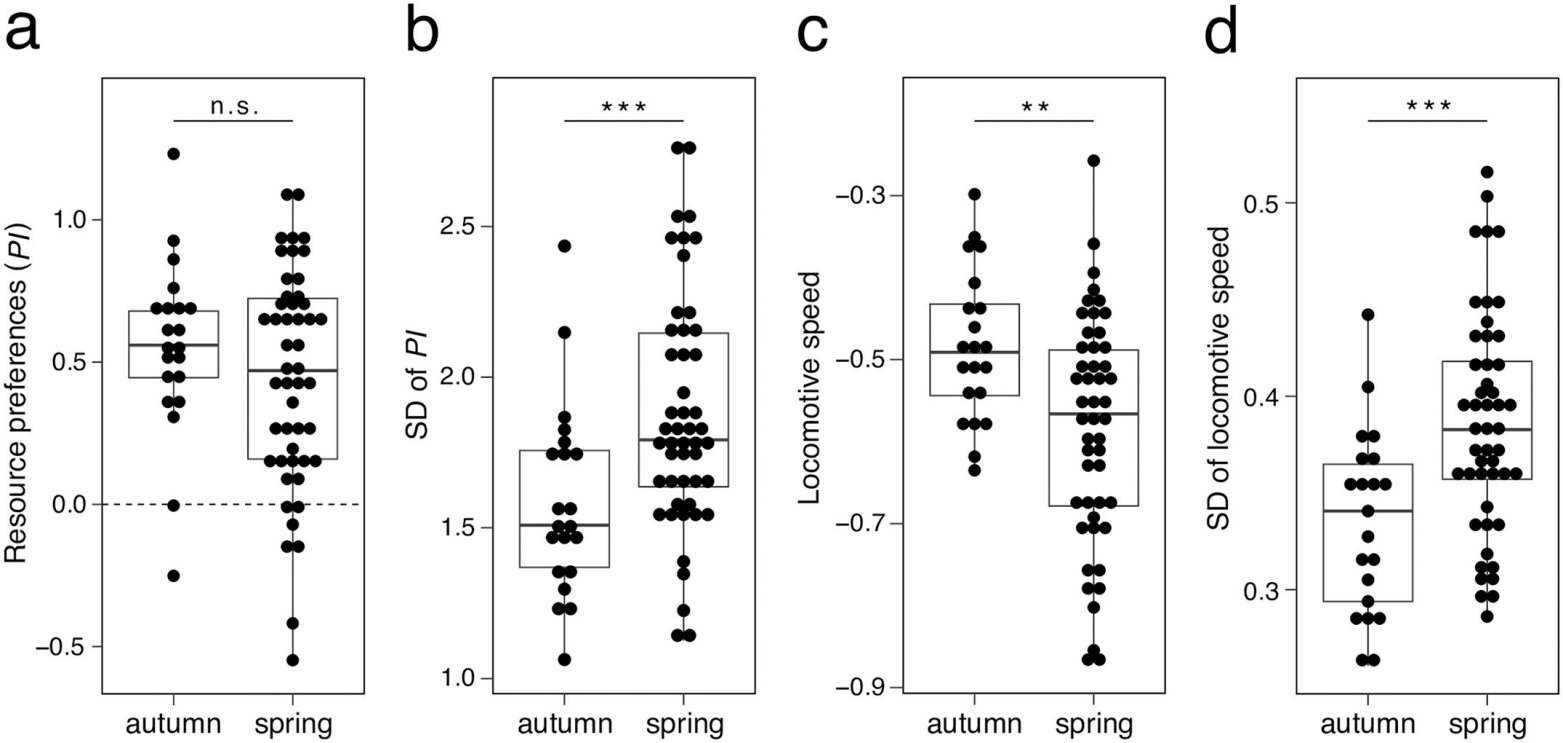
The seasonal differences in each trait mean and variance (*N* = 70). Welch two sample *t*-test: **P < 0.01, ***P < 0.001.

The mean resource preferences of the 70 isofemale lines of *D*. *lutescens* showed a bias toward orange, with a significant deviation from 0 (*df* = 69, *t* = 10.9, *P* < 0.001). Preference for orange was significantly predominant in some lines, while other lines exhibited no preference (Fig 2a). No lines exhibited a significant preference for grape. The *PI* varied significantly among the lines (*df* = 1, χ^2^ = 6.65, *P* = 0.0099), with a range from −0.55 to 1.23 for the smallest and largest lines, respectively. The heritability of preferences calculated through *PI* was *H*^2^ = 1.25%. The degree of individual variation for *PI* (SD) ranged from 1.06 in the smallest line to 2.78 in the largest line. While the mean *PI* did not vary significantly between isofemale lines of which founders were collected in spring and those in autumn (*df* = 46.3, *t* = 1.24, *P* = 0.22; Fig 3a), the degree of intraline variation for *PI* (SD) was significantly higher in spring lines than in autumn ones (*df* = 46.0, *t* = −3.19, *P* = 0.0025; Fig 3b).

Locomotive activity also exhibited significant variation among the lines, with the largest and smallest lines exhibiting locomotive speeds of 0.8 and 0.2 mm/s (−0.26 and −0.87 for log-transformed), respectively (*df* = 1, χ^2^ = 112, *P* < 0.001; Fig 2b). The heritability of locomotive activity was estimated as *H*^2^ = 11.0%. The SD of locomotive activity for each line ranged from 0.26 in the smallest line to 0.52 in the largest line. The %CV locomotive activity for each line ranged from 55.1% in the smallest line to 134% in the largest line. Locomotive activity was significantly higher in the autumn lines than spring ones (*df* = 56.1, *t* = 3.55, *P* < 0.001; Fig 3c). The degree of intraline variation in locomotive activity was significantly higher in spring lines in the autumn lines (*df* = 45.1, *t* = −3.87, *P* < 0.001; Fig 3d, *df* = 43.3, *t* = −3.24, *P* = 0.0023; S1 Fig).

**Fig 4:**
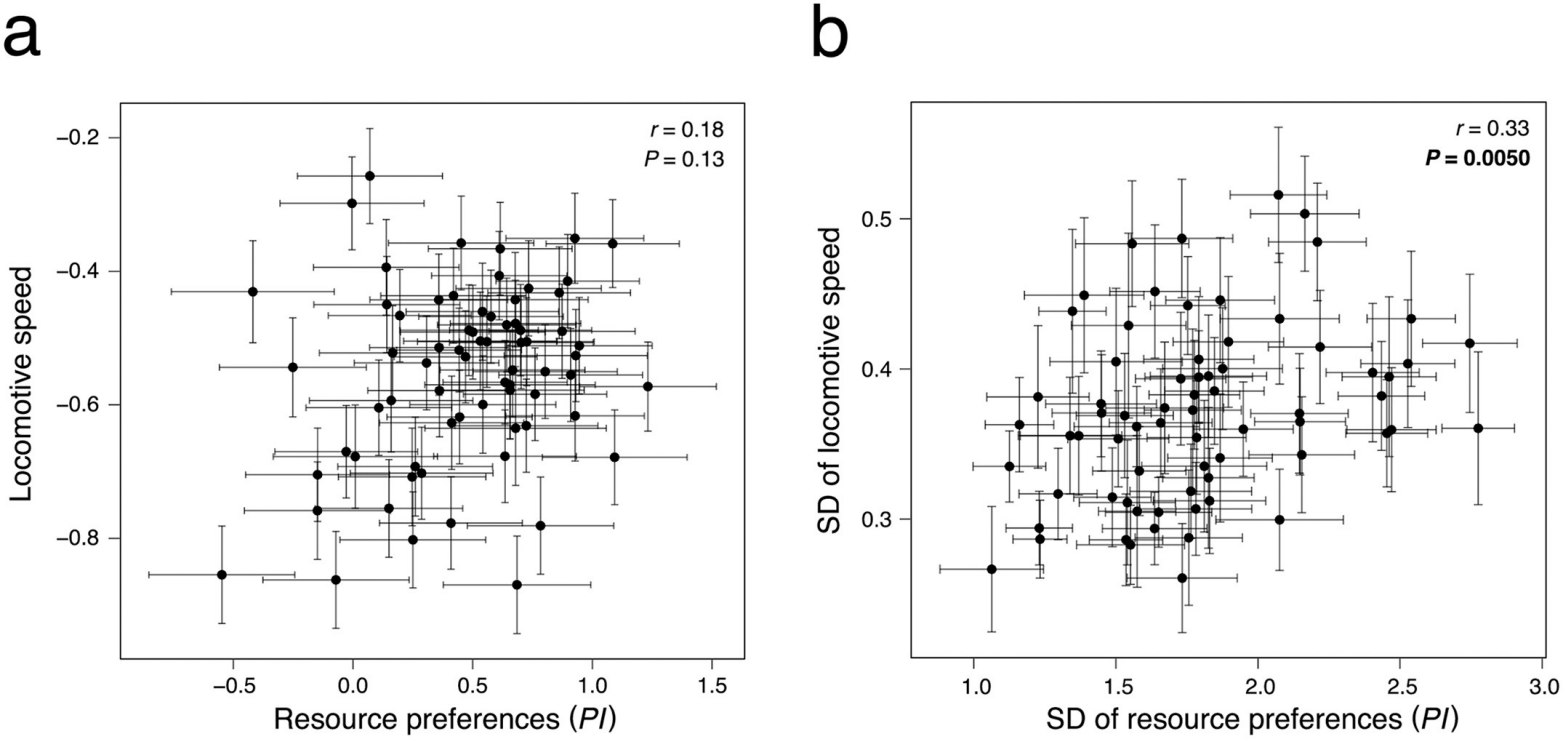
Correlations between mean locomotive speed and mean *PI* for each line (a), and correlations between intraline variation in locomotive speed and in *PI* for each line (b). Error bars represent standard errors. Each panel displays correlation coefficients and *P*-values determined using Spearman’s correlation test.

Correlation between mean resource preferences and mean locomotive speed was not significant (Fig 4a). However, a significant correlation was found between the intraline variation of resource preferences and that of locomotive speed (Fig 4b and S2 Fig), indicating that lines with higher intraline variation showed higher intraline variation in locomotive speed, vice versa. The covariance between mean resource preferences and mean locomotive speed was 0.012, and that between intraline variation of resource preferences and that of locomotive speed was 0.0073 and 0.047 for SD and %CV, respectively. In addition to the main analyses, the following correlation analyses were conducted to examine the possibility that the degree of variability depends on the mean phenotypic value of each line and to verify whether there is an artifact correlation between the variability of preference and of locomotive speed. No correlation was found between the mean and intraline variation of locomotive speed, while a negative correlation was found between the mean and intraline variation of resource preference (S3a and S3b Fig).

## Discussion

Despite the ecological significance of intraspecific niche partitioning [7–10], little research has focused on investigating the role of variability on such partitioning. In our study, we uncovered variation that may be driven by genetic differences between isofemale lines within the population as well as variability in foraging traits within each line. Notably, we found that the degree of variability might be heritable. This suggests its potential role in increasing phenotypic variation in resource utility among individuals within the population. However, we could not demonstrate the strong evidence for the heritable of variability due to missing the genetic background. Further studies are needed to explore the genetic underpinnings of variability in foraging traits in order to demonstrate the inheritance and evolution of this variability.

The intraline variation quantified in the present study may include measurement errors. If the quantified intraline variation resulted from measurement error, the noise in the two independent traits should not correlate with each other. However, we observed a significant positive correlation in the intraline variation of the two traits. This implies that the noise observed in locomotive activity and resource preferences is not solely due to measurement error but rather reflects variability in traits. Moreover, the significant positive correlation in the variability of the two traits suggests that a shared genetic basis likely governs the variability in both traits. Certain genetic factors may contribute to the occurrence of variability across multiple traits, as seen in plants and *Drosophila*, with the protein Hsp90 known to govern the variability in multiple traits [35, 36]. The evolution of genes that control the expression of such proteins may influence the degree of variability. The negative correlation between the variability and the mean of resource preference suggests that the dependency of variability on the mean could potentially result in an artifact correlation between the two variabilities. However, we did not find any correlation between the mean values of the two traits themselves, nor between the mean value and variability of speed. This indicates that the observed correlation between the two fluctuations is unlikely to be merely an artifact. The negative correlation between the variability and the mean of resource preference indicates that lines without line-level preference exhibited high variance in preference among individuals within lines. In the lines without line-level resource preferences, individuals might show highly variable preference or be exhibiting contrarian feeding choices to avoid resource competition with others. Further studies are needed to elucidate the mechanisms leading to phenotypic variation among individuals of the same line.

The degree of genetic variation within lines and larval density which was not controlled for in this study are potential explanations for the correlation between intraline variation in resource preferences and locomotor speed. In fact, many studies demonstrated that the experiences during larvae changed the phenotypes in adult flies [37–39]. Although we strived as much as possible to eliminate the influence of genetic and environmental factors on variation within a line, previous studies have shown that residual heterozygosity is maintained even within the lines inbred for 20 generations in *Drosophila* [40]. Therefore, currently, we cannot directly rule out the possibility that intraline variation quantified in our study partially reflects the degree of genetic variation within a line. We did not find a significant correlation between mean resource preferences and mean locomotive speed, suggesting that the genetic basis of these two traits is independent and encoded by different loci. If the intraline variation in our measure was due to residual heterozygosity, the extent of genetic variation at the two independent loci would coincidentally covary. While we cannot rule out this possibility, it is also possible that a common genetic basis underlies the degree of variability in both traits. On the other hand, the effects of environmentally induced variation may be caused by line-specific environmental condition, which are established by line-specific feeding behavior. At present, there is little evidence to refute the occurrence of variability due to environmental heterogeneity caused by larvae.

In the present study, the spring generation exhibited larger variability in both traits than the autumn generation, suggesting rapid seasonal evolution of variability. Residual heterozygosity is less likely to explain seasonal changes in intraline variation for each trait since the spring generation has been maintained for a longer period than the autumn generation. In *Drosophila*, the previous theoretical studies have shown that increasing the variability of traits is one of the adaptive strategies for fluctuating environmental conditions like seasonal changes [41]. However, at this time, it is not yet known whether the variability of foraging traits investigated in the present study was the result by seasonal selection.

In the present study, *D*. *lutescens* tended to prefer orange juice over grape juice. In *Drosophila melanogaster*, females are known to prefer citrus over grapes as an oviposition substrate [32]. The females of *D*. *lutescens* may also have preference for orange over grapes as an oviposition substrate. Resource distribution and abundance in the habitat could explain the biased preference for orange. Many species have been shown to change their resource preferences depending on their habitats [42, 43]. In the habitat where females (i.e., founders of the inbred lines) were collected in the present study, many citrus species have been planted. Resource selection observed in the present study might reflect adaptation to local habitat. Note that at this time we cannot rule out the possibility that sampling methodology led to a bias in resource preferences, since we used bait traps containing only bananas as a fruit source. On the other hand, previous experiment using olfactory stimuli suggests that females prefer grapes over orange as a resource [33]. This contrasting preference between resource and oviposition substrate is hypothesized to be the underlying factor contributing to the ambiguous preferences in each line. Further experimental studies, accurately understanding the resources used by this species depending on the population, are required to clarify the niche partitioning in *D*. *lutescens*.

Resource preferences and locomotive activity may directly or indirectly lead to intraspecific niche partitioning, which has crucial ecological implications, such as avoiding competition [44, 45]. The occurrence of intraspecific niche partitioning has been reported in various organisms [46, 47]. Alongside genetic variation, phenotypic plasticity and variability could also contribute to generating variation in foraging traits within a population, thereby mitigating resource competition among individuals. On the other hand, we did not quantify the contributions of genetic variation within the lines, the environment factors, and measurement error in our experiments. Therefore, it is difficult to evaluate the pure contribution of variability to total phenotypic variation and the relative contribution of genetic, environmental and stochastic effects on total phenotypic variation. Further experimental studies are required to clarify the contribution of variability in generating variation within a natural population and its effects on population dynamics.

## Supporting information

S1 Fig The seasonal differences between intraline variation in locomotive speed (%CV) and in *PI* for each line (*N* = 70). Welch two sample *t*-test: **P < 0.01.
(TIF)

S2 Fig Correlations between intraline variation in locomotive speed (%CV) and in *PI* for each line. Error bars represent standard errors. Each panel displays correlation coefficients and *P*-values determined using Spearman’s correlation test.
(TIF)

S3 Fig Correlations between the mean and intraline variation in resource preference (a) and locomotive speed (b). Error bars represent standard errors. Each panel displays correlation coefficients and *P*-values determined using Spearman’s correlation test.
(TIF)

S1 Table Foraging traits, females collected dates, replicates, and the *P*-values (from the binomial test) for each line. The SD and CV of foraging traits, as well as the *P*-values were corrected using jackknife estimation and the FDR, respectively.
(XLSX)
