# Supplementary figures and images for: Stochastic variation in foraging traits within inbred lines of Drosophila
Source: PLoS One. 2025 Jan 16;20(1):e0289864. doi: 10.1371/journal.pone.0289864 (PMC11737734; doi:10.1371/journal.pone.0289864)

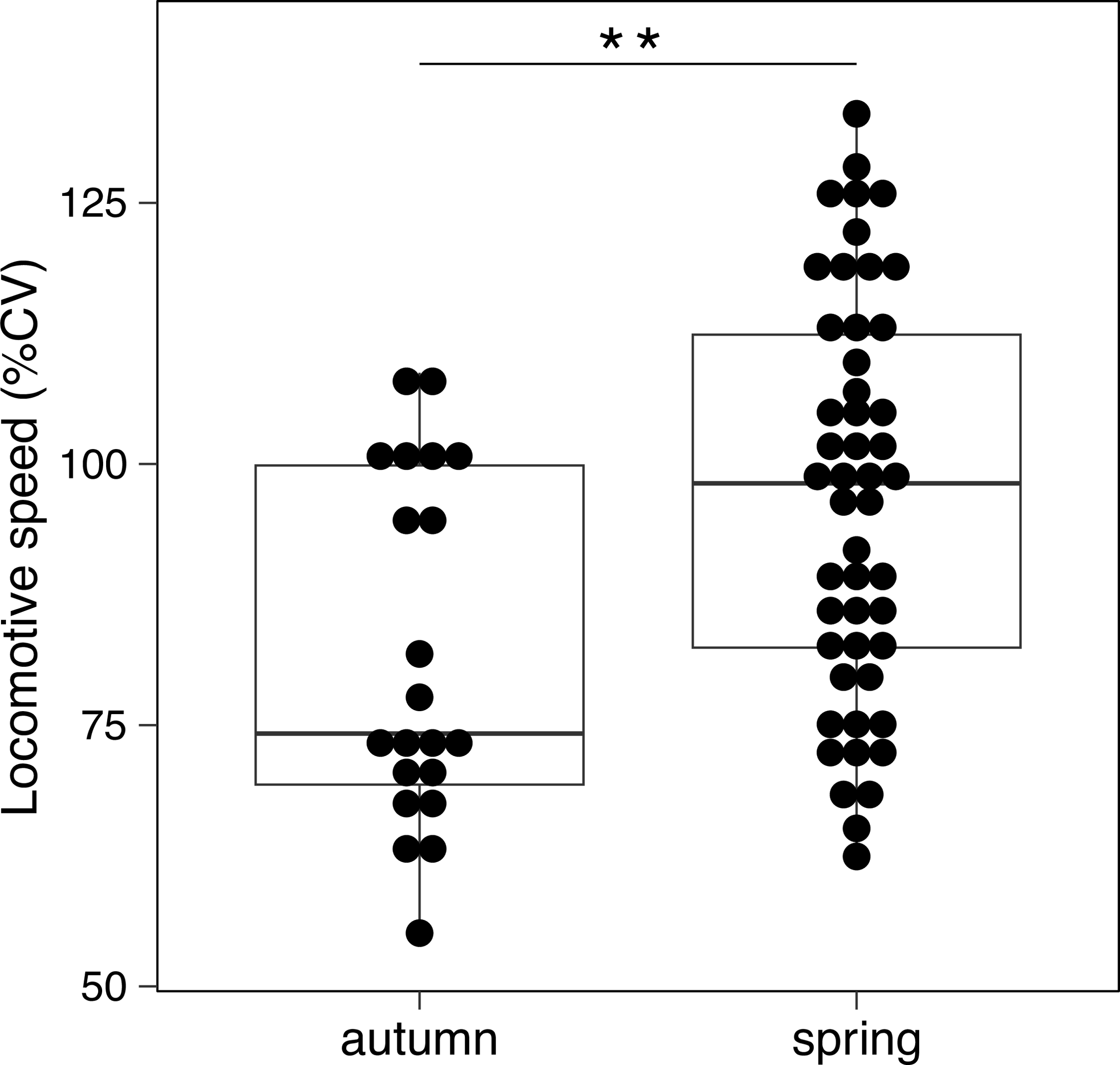

Supplement: S1 Fig — Welch two sample t-test: **P < 0.01. (TIF) [file pone.0289864.s001.tif]

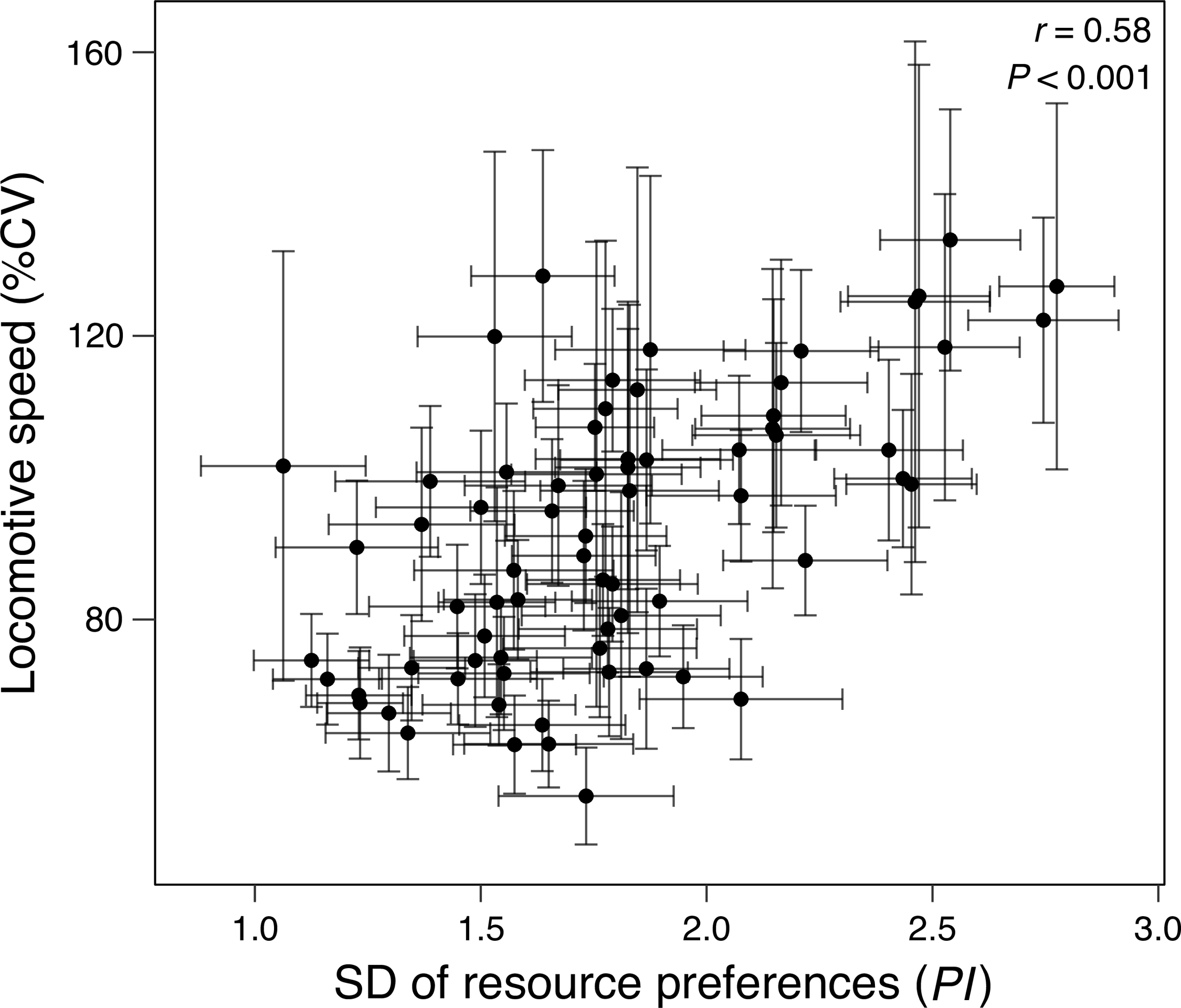

Supplement: S2 Fig — Error bars represent standard errors. Each panel displays correlation coefficients and P-values determined using Spearman’s correlation test. (TIF) [file pone.0289864.s002.tif]

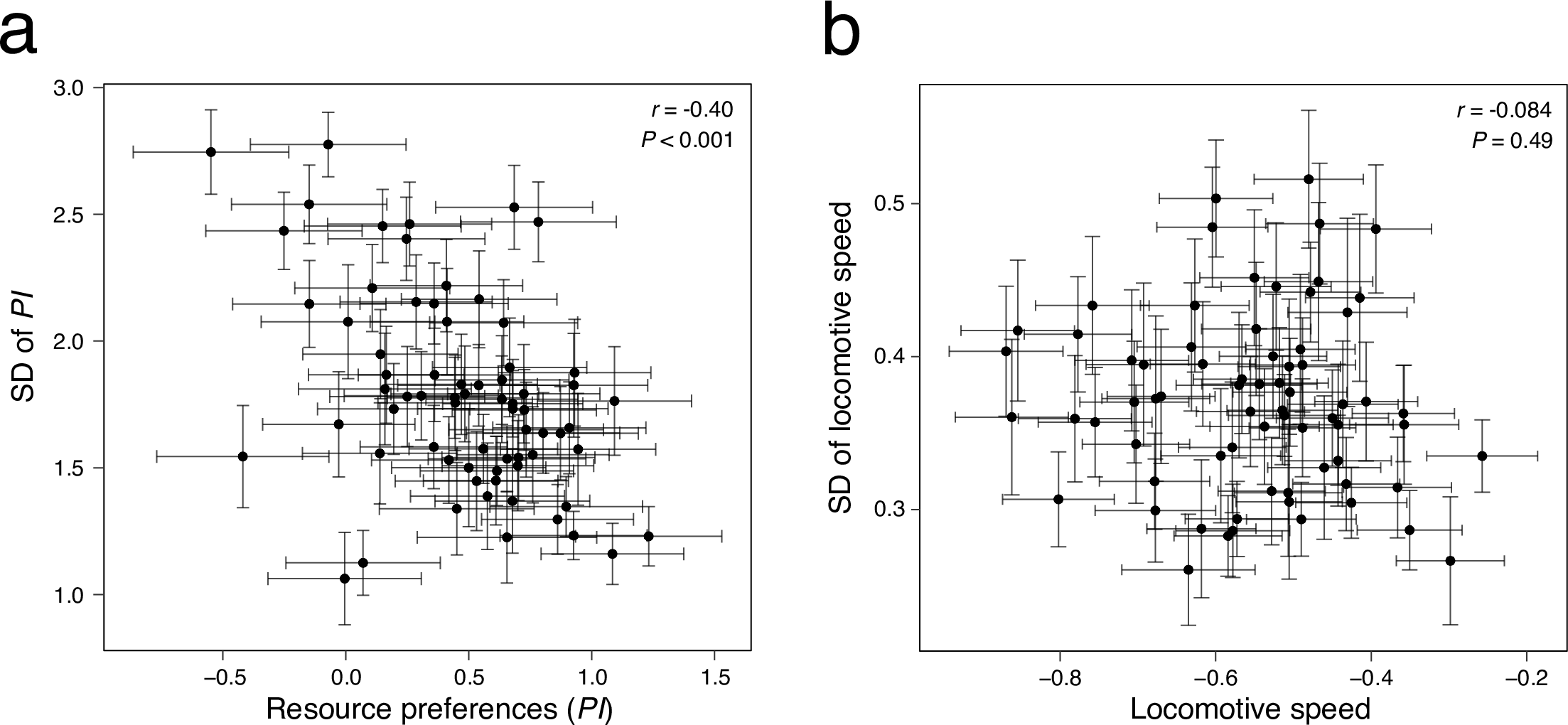

Supplement: S3 Fig — Error bars represent standard errors. Each panel displays correlation coefficients and P-values determined using Spearman’s correlation test. (TIF) [file pone.0289864.s003.tif]
